# Clinical value of lncRNA TUG1 in temporal lobe epilepsy and its role in the proliferation of hippocampus neuron via sponging miR-199a-3p

**DOI:** 10.1080/21655979.2021.2001904

**Published:** 2021-12-07

**Authors:** Chunlian Li, Xiaojing Zheng, Pingping Liu, Meilian Li

**Affiliations:** a Department of Pediatrics, Affiliated Hospital of Weifang Medical University, Weifang, China; b Sterile Supply Room, Affiliated Hospital of Weifang Medical University, Weifang, China; c Orthopedics and Rehabilitation Department, Weifang Traditional Chinese Hospital, Weifang, Chinag

**Keywords:** LncRNA TUG1, miR-199a-3p, proliferation, apoptosis, hippocampus neuron, temporal lobe epilepsy

## Abstract

Temporal lobe epilepsy (TLE) often occurs in childhood and is the most common type of epilepsy. Studies have confirmed that long non-coding RNAs (lncRNAs) can affect the progression of neurological diseases. This study explored the expression level of lncRNA TUG1 in TLE children and its clinical significance and investigated its role in hippocampal neurons. 86 healthy individuals and 88 TLE children were recruited. The expressions of lncRNA TUG1 and miR-199a-3p in serum were detected by qRT-PCR. Hippocampal neurons were treated with non-Mg^2+^ to establish TLE cell model. MTT assay and flow cytometry assay was used to detect the effect of lncRNA TUG1 on the proliferation and apoptosis of hippocampal neurons. A dual-luciferase reporter assay was done to confirm the target relationship. The expression of lncRNA TUG1 was increased in TLE children compared with the control group. The diagnostic potential was reflected by the receiver operator characteristic (ROC) curve, with the AUC of 0.915 at the cutoff value of 1.256. Elevated levels of TUG1 were detected in TLE cell models, and TUG1 knockout could enhance cell activity and inhibit cell apoptosis. MiR-199a-3p was the target of TUG1. Clinically, the serum miR-199a-3p levels showed a negative association with TUG1. LncRNA TUG1 may be a biomarker of TLE diagnosis in children, and can regulate hippocampal neuron cell activity and apoptosis via sponging miR-199a-3p.

## Introduction

Epilepsy is a chronic neurological disorder caused by cortical neuron abnormalities and synchronous firing [1–4]. Temporal lobe epilepsy (TLE) is a common chronic neurological disease in epilepsy [5]. TLE always occurs in children, with an average of 32 to 82 cases per 100,00. The onset of TLE is characterized by abnormalities in excitatory ion channels and ionic receptors [6]. The confirmation of the diagnosis of epilepsy often relies on active electroencephalogram (EEG), advanced neuroimaging, together with an appropriate medical history [7]. Currently, the main treatment for epilepsy is the chronic administration of anticonvulsant drugs (AEDs) [8,9]. TLE attack is accompanied by neuronal apoptosis, which seriously affects the nervous system function of children. And prevention of recurrent seizures is the main goal in the treatment of epilepsy. But the mechanism of TLE remains unclear.

Long non-coding RNAs (lncRNAs) are non-coding RNAs with a length of about 200 bp and can be involved in a variety of biological functions [10]. It has been studied in a variety of neurological disorders [11]. LncRNA taurine‐upregulated gene 1 (TUG1) is a regulator of many physiological and pathological processes [12]. It has been widely reported to be abnormally expressed in various human diseases, including neurological diseases [13,14]. For instance, a study has shown that TUG1 knockdown can prevent neurons from death to alleviate acute spinal cord injury [13]. In Parkinson’s disease, silencing TUG1 can inhibit SH-SY5Y cell apoptosis and reduce neuroinflammation, thus playing an effective protective role [15]. Notably, Parkinson’s is one of the neurodegenerative diseases, along with epilepsy. However, the role of TUG1 in TLE children and its mechanism is still limited.

Thereby, 88 children with TLE were recruited in the present study, and the expression changes of TUG1 were examined. Furthermore, the TLE cell model was established, and the role of TUG1 in TLE was also explored.

## Materials and methods

### Subject

174 children were recruited without the following conditions: (1) electrolyte disorder (2) metabolic disorder (3) acute brain disease (4) non-epileptic seizure events that mimic epilepsy. The subjects were divided into two groups: 86 healthy individuals (10.06 ± 3.07 years, male/female 48/38) and 88 children with TLE (9.72 ± 3.27 years, male/female 53/35). Peripheral blood of each subject was collected and centrifuged, then the serum samples were stored at −80°C.

The study protocol was approved by the Ethics Committee of Affiliated Hospital of Weifang Medical University, and written informed consent of each subject was obtained.

### Culture of hippocampal neurons

Hippocampal cells were collected from the newborn rats (Shanghai Animal Experimental Center)following previous studies [16]. The culture methods were as follows. The newborn rats were firstly anesthetized with sodium pentobarbital, and the hippocampus tissues were collected. Then the hippocampal cells were dissociated from the hippocampus using 0.5% trypsin at 37°C for 15 minutes. Subsequently, the DNase I treated warp elements were placed on the coated medium and cultured in a humidified incubator at 37°C and 5% CO_2_. Finally, the cells were transferred to the neurobasal medium.

### Establishment of epilepsy model

Hippocampal neurons of newborn rats were cultured in magnesium-free (non-Mg ^2+^) medium at 37°C for 3 h to induce epileptic activity.

### Cell transfection

Si-TUG1, miR-199a-3p mimic, miR-199a-3p inhibitor, and their negative control (si-NC,mimic-NC, inhibitor NC) were provided by Gene-Pharma (Shanghai, China). The cells were seeded into a 6-well plate at a concentration of 1 × 10^5^ cells/well. The cells were transfected the above sequences during the confluence of seeding cells were to 70–90%. Transfection was performed using Lipofectamine 2000 according to the manufacturer’s protocol.

### RNA extraction and Real-Time PCR

Total RNA was extracted using TRIZOL reagent (Invitrogen, Carlsbad, CA, USA). Reverse transcription was performed using the reverse transcription kit (Toyobo, Osaka, Japan). qRT-PCR was performed to detect gene expression using SYBR premix Ex Taq ^TM^. II commercial kit and the Applied Biosystems 7900 Real-Time PCR System. The relative gene expression was normalized to that of the internal control U6 or GAPDH according to the comparative delta CT (2^−ΔΔCt^) method.

### Cell viability assay

The cell viability was detected by MTT method according to the following steps. Cells were seeded in 96-well plates and cultured at 5% CO_2_ and 37°C for 3 days. Then 50 μl MTT solution was added to the plate and incubated for another three hours. Then, 150 ul DMSO (Sigma-Aldrich. Merck) was added to each well, and the absorbance was measured at 490 nm with a microplate reader.

### Cell apoptosis

Cell apoptosis was evaluated using an Annexin V-FITC Apoptosis Detection kit. The cells were washed with PBS, resuspended, and mixed with 5 μl Annexin V-FITC and PI staining solution in the dark for 5 minutes. The apoptosis rate was measured by FACS Cali bur flow cytometry (BD Biosciences).

### Dual-Luciferase reporter assay

MiR-199a-3p was predicted to be a candidate target gene of TUG1 by starbase, and then the relationship was verified using the luciferase reporter assay. The 3ʹ-untranslated region (UTR) of TUG1 was cloned into the luciferase reporter vector psiCHECK-2 (Promega Corporation) according to the manufacturer’s instruction. Briefly, 500 ng of each reporter construct [wild-type (WT) or mutant 3ʹ-UTR of TUG1 or the psiCHECK-2 vector] and miR-199a-3p mimic (5ʹ-ACAGUAGUCUGCACAUUGGUUA-3ʹ) or inhibitor (5ʹ- UAACCAAUGUGCAGACUACUGU-3ʹ) were co‑transfected into the cells. 48 hours post-transfection, the relative activity of luciferase was determined with a microplate reader (Molecular Devices, LLC). Renilla luciferase was used for normalization. Each sample was repeated three times.

### Data analysis

All data were analyzed using SPSS 18.0 software (SPSS Inc, Chicago, IL) and GraphPad Prism 7.0 software (GraphPad Software, Inc, USA). The data were presented as mean ± standard deviation (SD). The analysis methods were Chi-square test, Student’s T test or one-way ANOVA analysis of variance, and ROC curve. The difference was statistically significant when *P* < 0.05.

## Results

86 healthy individuals and 88 TLE cases were recruited, and the levels of TUG1 were detected using qRT-PCR. ROC curve was established for the diagnostic value assessment. In vitro, levels of TUG1 were regulated via cell transfection, and the role of TUG1 in neuron cell viability and apoptosis was explored. Then the target gene of TUG1 was predicted via bioinformatics, and the relationship was verified using luciferase activity assay.

### Clinical characteristics of the subjects

Table 1 summarized the clinical characteristics of healthy children and those with TLE. There were no statistically significant differences in age and sex distribution between healthy children and children with TLE (*P* > 0.05, Table 1). The results showed that the two groups had a good comparability.

**Table 1.** Baseline characteristics of the subjects

Characteristics	Healthy individuals (n = 86)	TLE children (n = 88)	*P* value
Mean (SD) age,years	10.06 (3.07)	9.72 (3.27)	0.506
Gender (male/female), n	48/38	53/35	0.555

Note: TLE, temporal lobe epilepsy; SD, standard deviation.

### TUG1 expression level in serum of subjects

**Figure 1.**
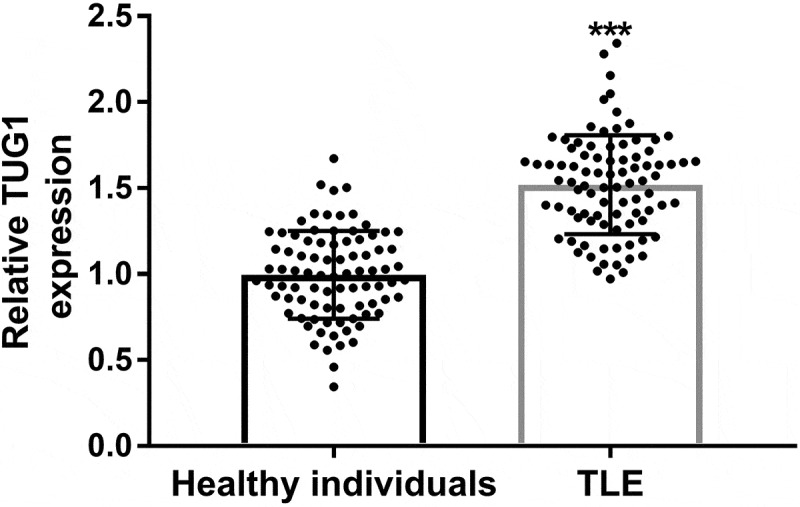
Serum expression levels of TUG1 in children with temporal lobe epilepsy (TLE) and healthy controls. The expression of TUG1 in TLE children was significantly increased. *** *P* < 0.001, compared with the healthy individuals

The expression of TUG1 was measured by qRT-PCR. The results showed that TUG1 expression level was significantly increased in TLE children compared with the control group (*P < 0.001*, Figure 1).

### Diagnostic value of TUG1 in TLE

**Figure 2.**
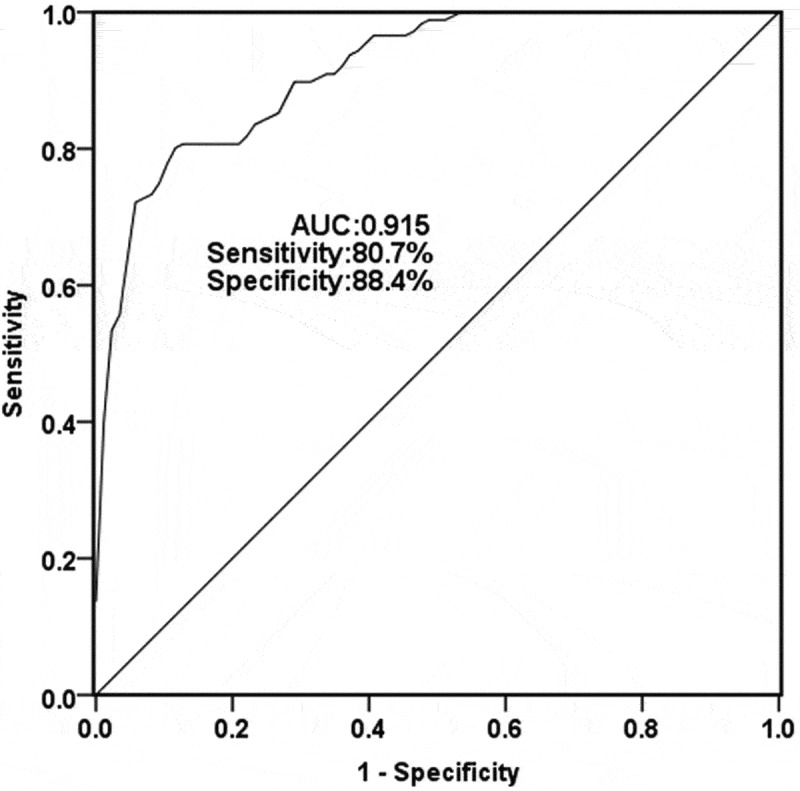
A receiver operating characteristic ROC curve was conducted to calculate the diagnostic ability of serum TUG1 for TLE. The area under the curve (AUC) for TUG1 was 0.915, with a sensitivity of 80.7% and specificity of 88.4% at the cutoff value of 1.256

As shown in Figure 2, when AUC was 0.915, the sensitivity and specificity were 80.7% and 88.4%, respectively, and the cutoff value was 1.256. The results showed that TUG1 might have diagnostic potential for TLE patients.

### Effects of TUG1 on hippocampal cells

**Figure 3.**
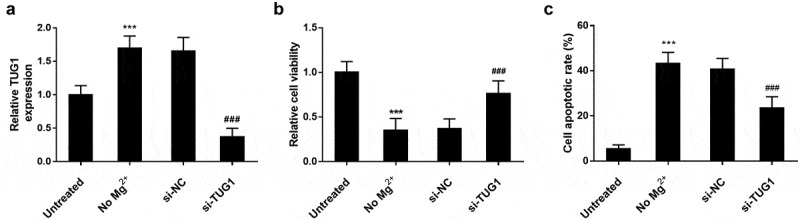
Effect of TUG1 on cell viability and apoptosis of hippocampal neurons. (a) primary hippocampal cells of newborn rats were cultured in the magnesium-free medium for three hours to simulate the symptoms of TLE in children. High expression of TUG1 was observed in cells treated in the magnesium-free medium compared with the untreated cells. Transfection of si-TUG1 led to a remarkable decrease in the expression level of TUG1. (b) No Mg^2+^ treatment reduced the cell viability, but the cell viability was promoted by knocking out TUG1. (c) No Mg^2+^ treatment promoted cell apoptosis, however, knocking out TUG1 inhibited TLE – induced apoptosis. *** *P* < 0.001, compared with untreated group; ^###^ *P* < 0.001, compared with no Mg^2+^ group

As shown in Figure 3(a), the transfection of si-TUG1 showed a significant impact on the expression level of TUG1, and a significant decrease was observed. As shown in Figure 3(b), MTT results showed that the viability of hippocampal cells in Mg^2+^ free medium was significantly lower than that in the untreated group. but the inhibitory effect on cell viability was significantly reversed after si-TUG1 transfection. As shown in Figure 3(c), the cell apoptosis was significantly promoted by Mg^2+^ free medium, while. si-TUG1 transfected reversed the effect (*P < 0.001*).

### The target of TUG1 was miR-199a-3p

**Figure 4.**
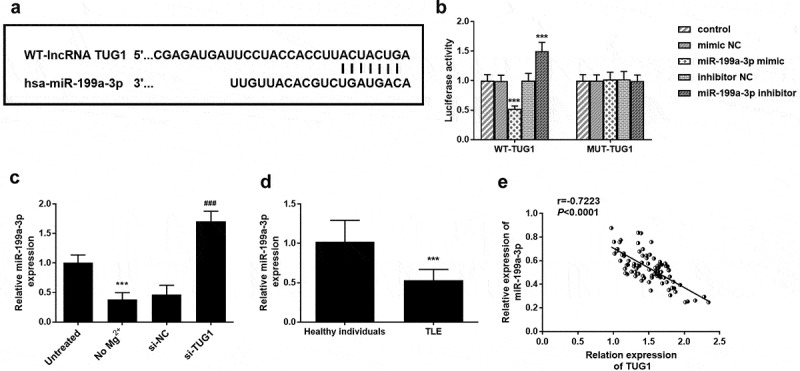
MiR-199a-3p was a direct target of TUG1. (a) the binding site of TUG1 in miR-199a-3p. (b) miR-199a-3p mimic significantly inhibited the luciferase activity of WT 3 ‘-UTR of TUG1, while the luciferase activity of miR-199a-3p inhibitor was the opposite. Furthermore, the transfection of miR-199a-3p mimic or miR-199a-3p inhibitor did not affect luciferase activity in the mutant group. (c) compared with the control group, the expression level of miR-199a-3p was significantly decreased in the no Mg^2+^ group and significantly increased in the si-TUG1group. (d) the expression level of miR-199a-3p was significantly reduced in children with TLE compared with healthy individuals. (e) the expression of TUG1 in TLE children was negatively correlated with miR-199a-3p level (r = −0.7223, *P* < 0.0001). *** *P* < 0.001, compared with the untreated group, control group, or healthy individuals. ^###^ *P* < 0.001, compared with No Mg^2+^ group

Figure 4(a) shows the binding sites of TUG1 with miR-199a-3p. Luciferase report results showed that miR-199a-3p inhibitors significantly increased the cell luciferase activity in the wild group, while miR-199a-3p mimic had the opposite effect (Figure 4(b)). The transfection of miR-199a-3p did not have any effect on the mutant group. As shown in Figure 4(c), the expression level of miR-199a-3p was significantly decreased in the cells treated with Mg^2+^, while the expression level of miR-199a-3p was significantly increased when transfected with si-TUG1. Subsequently, the expression level of miR-199a-3p in serum was detected. As shown in Figure 4(d), the expression level of miR-199a-3p was significantly reduced in children with TLE compared with healthy individuals. And the correlation between TUG1 expression and target miR-199a-3p in TLE children was also analyzed. The results showed that TUG1 expression was negatively correlated with miR-199a-3p expression in TLE children (Figure 4(e), r = −0.7223, *P* < 0.0001)

## Discussion

Epilepsy is a chronic nervous system disease that can be triggered by a variety of diseases, such as cerebrovascular disease, nutritional metabolic diseases, and brain damage after febrile convulsion. TLE is the most common type that affects children [17]. Large amounts of data indicate that lncRNA can be applied to epilepsy. For example, inhibition of H19 expression can protect selenium-induced hippocampal neuron damage and provide a new target for reducing epileptics-induced brain damage [18]. Therefore, no matter what kind of cause of epilepsy, understanding the regulatory mechanism of lncRNA is essential for the treatment of epilepsy, especially TLE. It is important to note that both Parkinson’s and TLE are neurodegenerative diseases [15]. In addition, studies have confirmed that TUG1 is overexpressed in Parkinson’s disease. It is speculated that TUG1 might have a potential role in TLE. As expected, elevated levels of TUG1 were measured in TLE children, which is consistent with our hypothesis. Recently, the clinical diagnosis of epilepsy is mostly by EGG and neuroimaging, but the clear diagnosis is mainly based on a detailed examination of the clinical manifestations and detailed medical history. Therefore, considering the abnormal expression of TUG1 in children with TLE, we further studied the diagnostic ability. ROC curve results showed that TUG1 had high sensitivity and specificity in children with TLE, which suggested the diagnostic potential of TUG1 for children with TLE.

Epilepsy is a neurodegenerative disease, which can cause different degrees of damage to hippocampal neurons and affect cognition and memory functions [19]. Hippocampal neuron apoptosis occurs at chronic stages of epilepsy, and it is necessary to promote neuron proliferation and prevent neuron apoptosis following epilepsy [20]. An increasing number of studies have found that lncRNA can affect the biological behavior of hippocampal neurons, thereby regulating the progression of a variety of diseases [21,22] For example, the study of Wang et al. confirmed that knocking out TUG1 can inhibit hippocampal neuronal apoptosis and reduce vascular cognitive impairment [23]. Cao et al. found that silencing TUG1 can reduce the neurotoxicity of rat hippocampal neurons induced by ketamine [24]. In this study, we investigated the role of TUG1 in restoring hippocampal neuronal activity and preventing neuronal cell death. The results showed that silencing TUG1 could increase the viability of hippocampal neurons and inhibit their apoptosis. Interestingly, in a model of Alzheimer’s disease, TUG1 silencing can increase neuron cell survival and inhibit apoptosis of hippocampal neurons [25]. The findings were consistent with our research results. Although the current study confirmed the role of TUG1 in TLE cell models, it will be interesting and necessary to verify the results in the TLE animal models.

As is known, lncRNAs act as promoting factors or suppressing elements in progress diseases by sponging specific sequences of target miRNAs. MiRNAs are a key regulatory molecule in cells that controls protein levels [26]. So far, more than 100 microRNAs have been found in the hippocampus that can regulate and control TLE. In Ewing’s sarcoma, the target regulatory interaction between miR-199a-3p and TUG1 is confirmed [27]. Increasing evidence has revealed the important role of miR-199a-3p in neurological diseases [28–30]. MiR-199a-3p is reported to play a protective role in neuron cells in a variety of diseases [14,31]. With the consideration of the finding, the role of miR-199a-3p in TLE attracts our concern. The target relationship between TUG1 and miR-199a-3p was confirmed via luciferase activity. Clinically, low levels of miR-199a-3p were detected in TLE patients, which was negatively correlated with the levels of TUG1. In a study about Parkinson’s disease, miR-199a-3p overexpression can protect against the disease progression via inhibiting neuron cell apoptosis and promoting cell proliferation [28]. In light of the target relationship of miR-199a-3p with TUG1, we speculated that miR-199a-3p might be involved in the regulatory role of TUG1 in TLE.

## Conclusion

In conclusion, TUG1 was abnormally expressed in TLE children. TUG1 silencing can inhibit apoptosis and promote the viability of the hippocampal cell. The findings might enrich the diagnostic methods of TLE children, and can provide a crucial basis for the future TLE therapeutic strategies.

## Highlights

- lncRNA TUG1 was identified to be upregulated in children with TLE.
- Knockdown of lncRNA TUG1 inhibited the apoptosis of hippocampal cells.
- lncRNA TUG1 may be a promising biomarker for the diagnosis of children TLE
- LncRNA TUG1 may be involved in the progression of TLE via sponging miR-199a-3p.
